# An anatomy-guided multi-vector thread lifting strategy for nasolabial fold correction: Technique refinement and clinical outcomes in a 22-patient case series

**DOI:** 10.1016/j.jpra.2026.04.013

**Published:** 2026-04-30

**Authors:** Gi-Woong Hong, Song-Eun Yoon, Jong Keun Song, Jin-Hyun Kim, Danilo Augusto Teixeira, Roshan Ravindran, Kyu-Ho Yi

**Affiliations:** a Samskin Plastic Surgery Clinic, Seoul, Korea; b Brandnew Aesthetic Surgery Clinic, Seoul, Korea; c Pixelab Plastic Surgery Clinic, Seoul, Korea; d You and I Clinic, Seoul, Korea; e Clinica Peli, Goiania, Goias, Brazil; f Roshan Ravindran Klinik, United Kingdom

**Keywords:** Rhytidectomy, Facial aging, Threads, Nasolabial fold, Minimally invasive, Surgical procedures

## Abstract

**Background:**

The nasolabial fold (NLF) is a key aesthetic concern in facial aging. Thread lifting offers a minimally invasive alternative to surgery but requires precise anatomical planning to ensure safety and efficacy.

**Objectives:**

To describe an anatomically informed, multi-vector thread lifting technique for NLF correction and evaluate clinical outcomes in 22 patients.

**Methods:**

Between March 2020 and December 2022, 22 patients (17 female, 5 male; mean age 47.2 years) underwent NLF correction using barbed threads for structural lifting and monofilaments for superficial volumization. Thread types and vectors were selected based on NLF subtype, skin thickness, and facial anatomy. Outcomes were assessed using standardized photographs and the Global Aesthetic Improvement Scale (GAIS) over a follow-up period of 6–18 months. The number and type of threads used in each patient were determined individually based on anatomical assessment and fold characteristics, rather than a fixed protocol, to allow personalized optimization of outcomes.

**Results:**

At 6 months, 90.9% of patients showed "improved" or "much improved" results. At 12 months, this remained in 77.8% of those followed. Type II NLFs showed strong initial results but greater regression over time, while Type III folds demonstrated better long-term stability. Complications were minor and self-limiting, with no serious adverse events.

**Conclusions:**

This technique offers a safe, tailored, and effective approach for minimally invasive NLF correction when guided by anatomical principles.

*Evidence level:* V

## Introduction

The nasolabial fold (NLF) is a prominent aesthetic concern, often deepening with age due to cumulative effects of midfacial volume loss, skin laxity, and gravitational descent of soft tissues. Traditional surgical approaches, while effective, may be excessive or undesirable for patients seeking minimally invasive alternatives. Thread lifting has emerged as a promising option, offering mechanical suspension of ptotic tissue with minimal downtime. However, achieving predictable and natural-looking results in the NLF region remains technically challenging due to its anatomical complexity, including the presence of facial retaining ligaments, mobile soft tissue interfaces, and proximity to critical vascular structures.^1^, ^2^, ^3^, ^4^, ^5^, ^6^, ^7^

In this study, we describe an anatomically informed, multi-vector thread lifting technique specifically tailored to address the NLF. The procedure utilizes a combination of barbed threads for structural repositioning and monofilaments for superficial volumization and dermal support. Thread selection and vector planning are adapted to individual facial types and NLF classifications, taking into account variations in malar prominence and paranasal volume. The technique emphasizes safe cannula trajectories that avoid key vascular structures such as the facial artery, while maximizing engagement of ligamentous anchor points including the medial and lateral maxillary ligaments. Vascular considerations and procedural depth transitions are integral to both the safety and longevity of the outcome.^1^, ^2^, ^3^, ^4^, ^5^, ^6^, ^7^

A total of 22 patients were treated using this method, with follow-up ranging from 6 to 18 months. Results demonstrated consistent improvement across NLF types, though with variable durability depending on individual anatomical features and soft tissue quality. By combining anatomical precision with a tailored thread strategy, this approach offers a reproducible and safe option for minimally invasive correction of nasolabial folds. The purpose of this manuscript is to present the technical protocol, clinical outcomes, complication profile, and anatomical rationale underlying this technique.

### Thread types and specific applications

Thread selection was performed using a structured, anatomy-based approach considering nasolabial fold type, skin thickness, and degree of tissue laxity. In general, barbed threads were selected for structural lifting in patients with ptosis-dominant folds (Type II and III), while monofilament or braided threads were used for superficial volumization and fold attenuation in volume-deficient or fine-line dominant cases (Type I). In patients with mixed characteristics, a combination approach was applied to address both lifting and volumization components.

**Figure 1:**
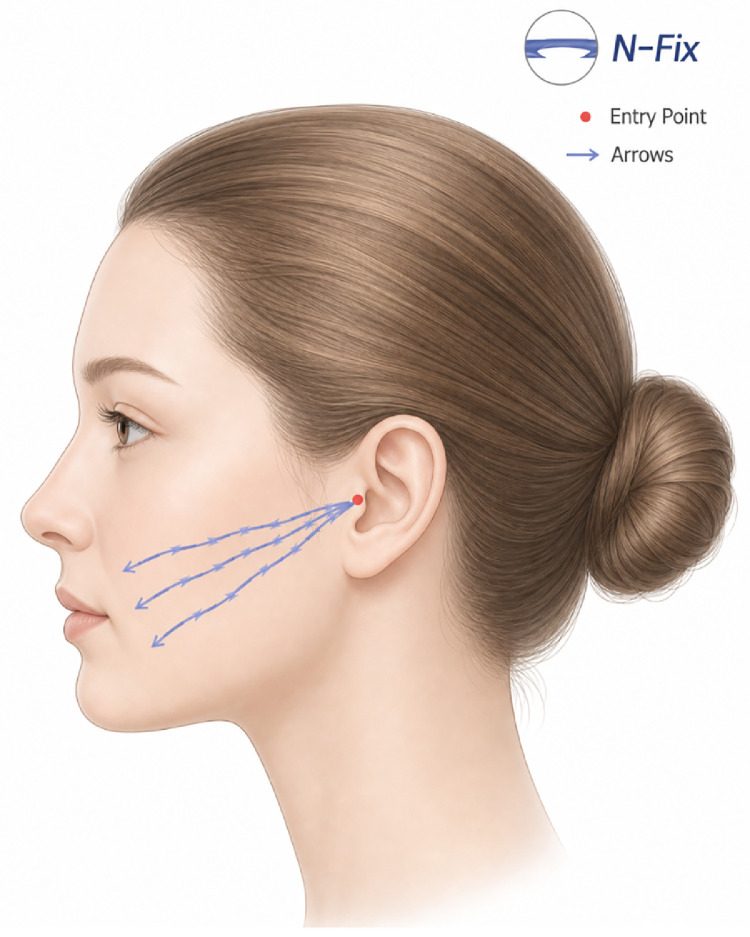
Optimal entry points and vector direction for barbed thread lifting of the nasolabial fold. A technical illustration demonstrating the recommended trajectory for barbed thread placement via Lore’s fascia, with depth adjustments as the cannula transitions from the lateral to the anterior face.

A multidirectional barbed absorbable thread (polydioxanone-based) such as N-Fix (N-finders, Korea) is inserted through the entry point for mass tissue fixation. The thread gauge is selected based on the patient’s skin thickness and the degree of tissue laxity (Figure 1).

**Figure 2:**
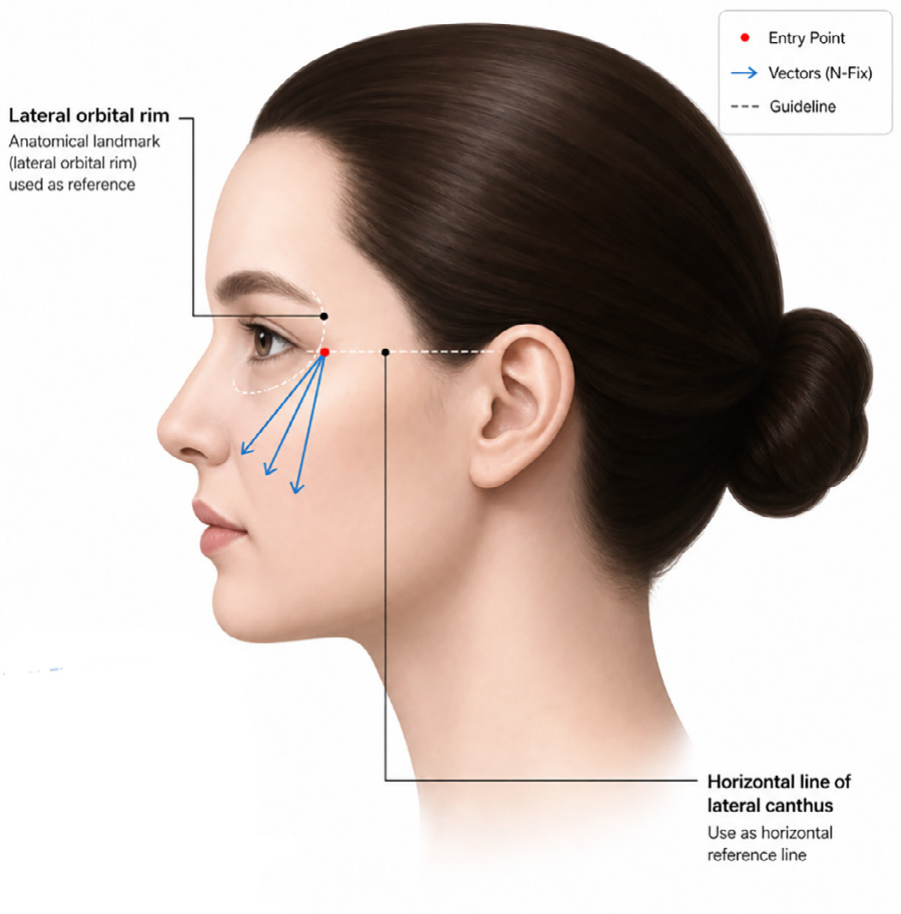
Entry points and direction for barbed thread lifting in the medial nasolabial region. A procedural diagram showcasing the periorbital approach for treating the alar base area, particularly in patients with prominent malar eminences, using NFix—threads.

This selection is typically preferred in patients with moderate laxity or thinner skin. Occasionally, a prominent band-like bulge may be observed at the nasolabial fold’s origin, where the alar base meets the cheek. In such cases, simply lifting the mid-portion of the fold may inadequately address the laxity of skin and soft tissues at this initial segment. 1–7 Direct traction on the paranasal area is necessary, but in patients with prominent malar eminences, which are frequently observed in East Asian patients in clinical practice, approaching from the preauricular region may result in cannula trajectory issues similar to those encountered with temple hairline entry points. In these instances, an approach analogous to anterior malar lifting through the lateral orbital thickening is advisable. N-Fix can be inserted through a periorbital entry point, bypassing the malar eminence to directly engage and reposition the ptotic fat layer near the alar base toward the periorbital region. The basic technique and tissue plane are consistent with those described for anterior malar lifting (Figure 2).

Post-procedurally, tissue tightening is observed, along with improved volumetric distribution. For fine residual fold lines resembling scar-like creases at the cheek-paranasal junction, volumizing monofilaments such as N-Scaffold (N-finders, Korea) can be employed to ameliorate these lines and enhance skin firmness.

In cases where fold attenuation is the primary goal without significant paranasal hollowing, N-Scaffold can be inserted slightly medial and parallel to the fold line. The entry point should be created inferiorly, accounting for the thread length. After needle puncture, the thread is inserted into the subcutaneous tissue superior to the orbicularis oris muscle, advancing from inferior to superior along the fold line.

**Figure 3:**
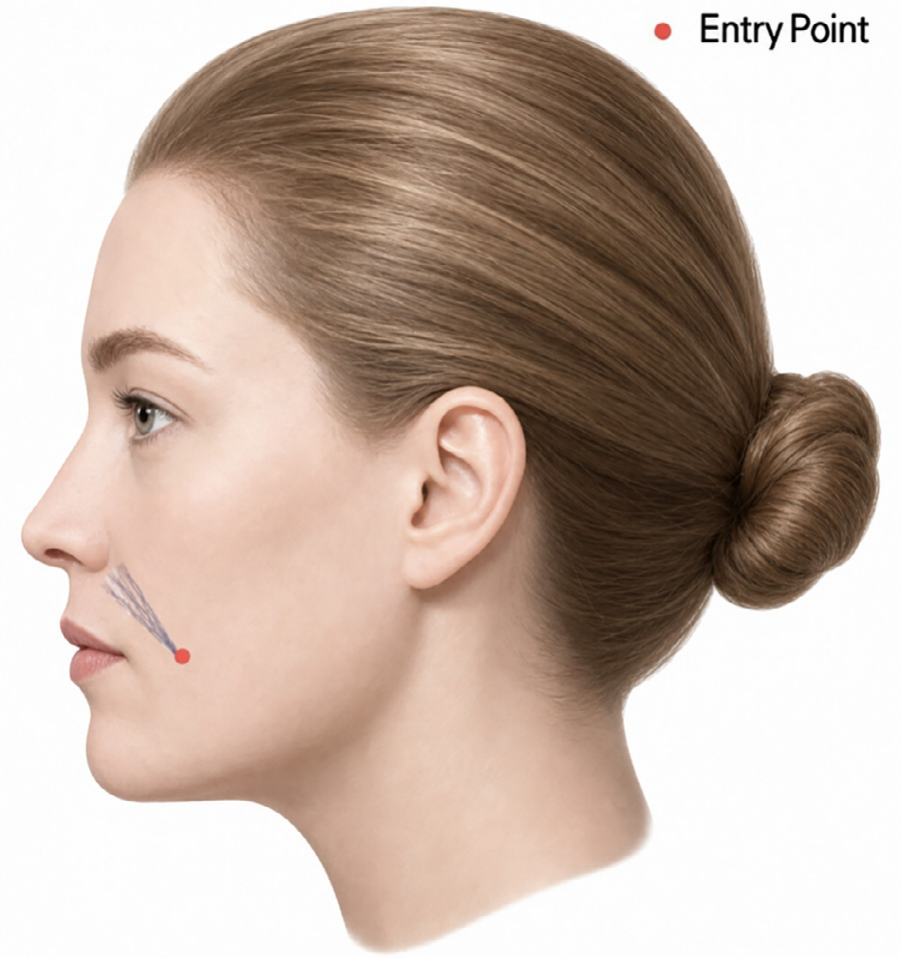
Entry points and direction for braided threads targeting the nasolabial crease. A technical illustration outlining the safe insertion sites for N-Scaffold monofilaments, positioned slightly medial to the fold line to minimize the risk of vascular injury while optimizing fold attenuation.

In over half of Koreans, the facial artery, including the branching point of the superior labial artery, courses superficially above the orbicularis oris muscle along the nasolabial fold, posing a risk of vascular injury during needle puncture or thread insertion. As the facial artery frequently follows the fold line, creating entry points a few millimeters away from the fold, as illustrated, can help mitigate the risk of needle-induced bleeding (Figure 3).

In this configuration, even in the absence of a single rigid fixation point, lifting is achieved through distributed tension across the superficial soft tissue layers. The braided structure of the thread allows engagement of multiple fibrous septa within the subcutaneous plane, creating a supportive network that stabilizes and attenuates the fold. This mechanism provides a subtle but clinically effective lifting and smoothing effect.

Maintaining a consistent depth and gentle advancement of the cannula is crucial not only for minimizing vascular injury risk but also for achieving a smooth, even post-procedural skin surface.

### Clinical experience and outcomes

Between March 2020 and December 2022, a total of 22 patients (17 female, 5 male; age range 34–65 years, mean 47.2 years) underwent nasolabial fold thread lifting using the described technique. The follow-up period ranged from 6 to 18 months (mean 11.5 months).

Nasolabial folds were categorized into three types based on dominant anatomical characteristics. Type I represents volume-deficient folds primarily related to midfacial fat atrophy. Type II corresponds to ptotic folds caused by soft tissue descent and ligamentous laxity. Type III reflects structurally deep folds associated with skeletal support deficiency and fixed crease formation.

In this series, 5 patients (22.7%) presented primarily with Type I folds, 10 patients (45.5%) with Type II, and 7 patients (31.8%) with Type III. However, 16 patients (72.7%) showed overlapping features of multiple types, underscoring the multifactorial nature of nasolabial fold formation.

**Figure 4:**
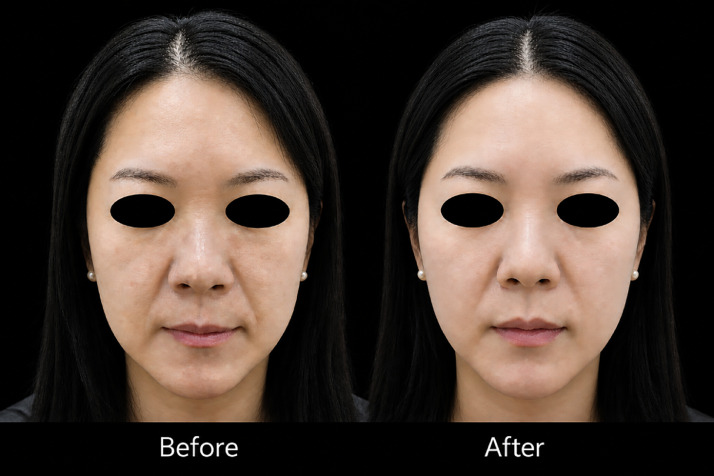
Representative clinical case of nasolabial fold correction. The patient was treated using a combination of barbed threads for structural lifting and monofilament threads for fold attenuation based on individual anatomical assessment. The pre-treatment image demonstrates a pronounced nasolabial fold, while the post-treatment image shows improved contour and reduced fold depth.

Aesthetic outcomes were assessed using standardized photography and the Global Aesthetic Improvement Scale (GAIS). At 6 months, 20 patients (90.9%) showed "improved" or "much improved" results. By 12 months, this percentage decreased to 77.8% of the patients followed to that point (18 patients) (Figure 4).

The longevity of results varied by nasolabial fold type, with Type II showing the most dramatic initial improvement but also the most significant regression over time. Type III folds showed more gradual improvement but better long-term stability. These observations guided our approach to thread selection and placement depth for different fold types.

### Complications and management

Thread lifting procedures may be associated with various complications that practitioners should be prepared to address. In our series of 22 patients, we observed the following complications.

### Minor complications

In our series of 22 patients, minor complications were relatively common but generally self-limiting. Mild bruising was the most frequent, occurring in 8 patients (36.4%) and typically resolving within 5–7 days without intervention. Edema affecting the treated area was noted in 5 patients (22.7%), with resolution generally occurring within 3–5 days and often responding well to cold compresses. Temporary sensory changes, including numbness or hypersensitivity along the thread path, were reported by 3 patients (13.6%). These sensory alterations resolved completely within 3 weeks in all cases and did not require specific treatment beyond patient reassurance.

Mild asymmetry was observed in 2 patients (9.1%) during follow-up assessments. Among these, both cases required minimal thread adjustment or additional thread placement to achieve better symmetry. Dimpling or surface irregularities occurred in 2 patients (9.1%), with one resolving spontaneously within 2 weeks as initial tissue edema subsided, while the other required gentle massage therapy to smooth out persistent irregularities.

### Major complications

Major complications were rare in our patient series. Thread extrusion occurred in one patient (4.5%), appearing after 5 weeks following the procedure at the entry point near the tragus. This case required thread removal and antibiotic prophylaxis, with subsequent healing without further complications. One patient (4.5%) developed a small hematoma following inadvertent vessel injury during needle puncture. This resolved with compression but resulted in more pronounced bruising that persisted for approximately 3 weeks.

No cases of facial nerve injury, infection, or granuloma formation were observed in our series, which we attribute to careful technique and proper patient selection. No patients reported persistent pain along the thread path.

### Prevention and management strategies

Careful cannula insertion through an entry point at the tragus-cheek junction, penetrating and advancing deep to the SMAS layer, allows safe thread progression in the lateral face without risking injury to the superficial temporal artery or premasseteric branch. While the facial artery typically courses parallel to the nasolabial fold, traversing above and below the mimetic muscles in this region, cannula-based thread lifting generally poses minimal risk if performed gently. However, needle-based monofilament insertion for fold attenuation requires particular caution due to the increased risk of vascular injury.^8^, ^9^, ^10^, ^11^

As the cannula transitions from the lateral to the anterior face, the procedural plane should be adjusted to engage the lax tissue superior to the nasolabial fold with the barbed thread, with the intention of anchoring it toward the anterior tragal region. It is crucial to ensure that the threads engage the ligamentous structures supporting the skin and tissues superior to the nasolabial fold, specifically the medial and lateral maxillary ligaments. This engagement facilitates the effective mobilization of the attached lax tissues toward the ear and enhances the longevity of the repositioning.

## Discussion

Our clinical experience with 22 patients demonstrates several advantages over traditional face lifting procedures for appropriate candidates. The minimally invasive nature significantly reduces recovery time and associated risks compared to surgical interventions. The ability to target specific anatomical issues without affecting uninvolved areas allows for more natural-looking results and the potential for combination with other minimally invasive procedures.

Despite these advantages, limitations must be acknowledged. The results, while significant, are generally less dramatic and shorter-lasting than surgical face lifting. The technique is highly operator-dependent, requiring thorough anatomical knowledge and technical precision. Outcome variability is considerable based on patient tissue characteristics, with thinner skin showing more immediate but potentially less durable results. Additionally, the follow-up period in this study was relatively short (maximum 18 months), which limits the ability to fully assess long-term durability of the results. Despite variability in individual anatomy, the use of a structured, anatomy-based approach enhances procedural reproducibility and may facilitate adoption by clinicians with appropriate anatomical training.

## Statement of human and animal rights or ethical approval

This study was conducted in accordance with the Declaration of Helsinki.

## Informed consent

Patient consent for photo publication: Written informed consent was obtained from all patients for the use and publication of clinical photographs and any potentially identifiable images in this article.

## Financial disclosure

There is no financial disclosure to report.

## Author contributions

Conceptualization: **Gi-Woong Hong, Kyu-Ho Yi, Song-Eun Yoon, Jong Keun Song, Jin-Hyun Kim, Danilo Augusto Teixeira, and Roshan Ravindran.** Writing-original draft preparation: **Gi-Woong Hong.** Writing-review and editing: **Gi-Woong Hong, Kyu-Ho Yi, and Song-Eun Yoon.** Visualization: **Gi-Woong Hong and Kyu-Ho Yi.** Supervision: **Gi-Woong Hong and Kyu-Ho Yi.** All authors have reviewed and approved the article for submission.

## Declaration of competing interest

The authors declare that they have no conflicts of interest to disclose.
